# A Case of Spontaneous Multivessel Coronary Artery Spasm That Underwent Stent Implantation Accompanying ST Segment Elevation on Inferior Electrocardiographic Leads

**DOI:** 10.1155/2016/7049748

**Published:** 2016-05-08

**Authors:** Nuray Kahraman Ay, Muharrem Nasifov, Ömer Goktekin

**Affiliations:** Department of Cardiology, Bezmialem Vakif University, Fatih, 34093 Istanbul, Turkey

## Abstract

Coronary artery spasm is usually defined as a focal constriction of a coronary artery segment, which is reversible, and causes myocardial ischaemia by restricting coronary blood flow. A coronary spasm may rarely compromise all three epicardial arteries simultaneously. We present a case of severe coronary spasm afflicting all coronary arteries accompanying an ST segment elevation in leads D2-D3 and aVF.

## 1. Introduction

Coronary artery vasospasm is an important cause of myocardial ischaemia. Myocardial infarction can occur in severe or prolonged ischaemia. Chest pain after successful percutaneous coronary intervention constitutes a considerable problem and is potentially life threatening when myocardial ischaemia occurs. Such pain indicates the presence of residual coronary stenosis, acute occlusion, coronary spasm, or myocardial infarction. The management of each type of patient involves repeat coronary angiography and additional interventions. Coronary angiography can clearly reveal coronary artery stenosis or acute occlusion, whereas coronary spasm is difficult to demonstrate with routine angiography.

## 2. Case Report

A 64-year-old man was admitted to the emergency department with squeezing chest pain. He was a nonsmoker with a history of hypertension, diabetes, and documented coronary artery disease. Over the preceding 8 months, he had undergone two coronary angiography procedures at another hospital. A 40% stenosis was discovered in the mid left anterior descending (LAD) artery, 90% stenosis was detected in the first diagonal and septal arteries, 40% stenosis was found in the second diagonal, 70% was found in the mid left circumflex (Cx), 98% diffuse stenosis was found in the intermediary artery, and 40% stenosis was found in the right coronary artery on the previous angiography report. Fractional flow reserve (FFR) of the Cx lesion was 0.79 and a 3.5 × 14 mm drug eluting stent was employed. Due to repeated similar complaints, angiography was performed again 5 months later at the same hospital. The angiographic results showed similar lesions but no instent stenosis. Drug therapy was administered to the patient, such as beta blocker, aspirin, angiotensin-converting-enzyme inhibitor, clopidogrel, and statin. The clinical examination was unremarkable, and there was no peculiarity on the electrocardiogram (ECG) in our emergency department. Troponin I was not elevated (0.045 ng/mL). However, treatment was commenced for presumed acute coronary syndrome due to his angina and cardiac history.

**Figure 1:**
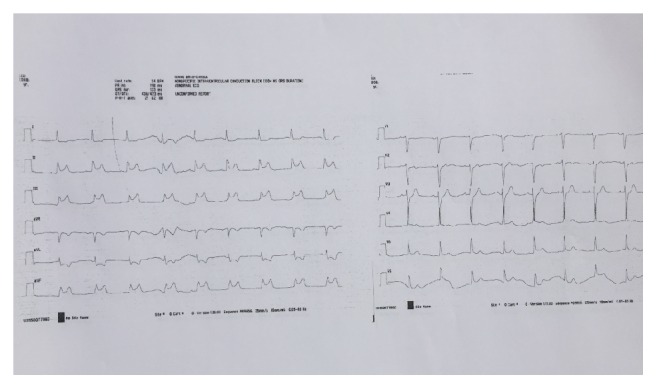
ST segment elevation on inferior electrocardiographic leads.

Two hours after admission, the chest pain recurred and the ECG exhibited evidence of an evolving ST segment elevation in the inferior leads (Figure 1). He was transferred immediately to the angiography unit for urgent angiography.

### 2.1. Angiography Findings

**Figure 2:**
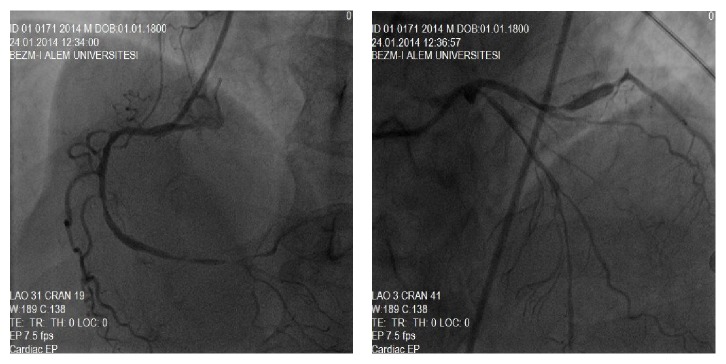
Multiple diffuse narrowing on right coronary artery, left anterior descending artery, and circumflex artery.

**Figure 3:**
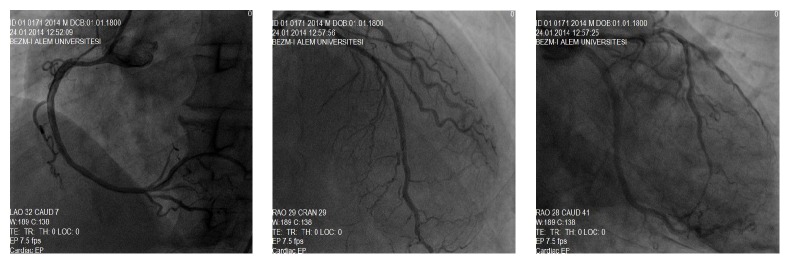
RCA and left system arteries after intracoronary nitroglycerin injection.

We suspected a subacute stent thrombosis of the Cx artery; thus, we visualised the right coronary artery (RCA) first. We observed a 99% lesion before the crux and diffuse mild narrowing from proximal to mid segment. Diffuse significant narrowing was detected in the LAD artery and both proximal and distal to the stent in the Cx artery, but no instent stenosis was detected (Figure 2). An intracoronary nitrate injection was not considered, in accordance with known treatments for coronary artery disease. We thought that the subtotal lesion in the distal RCA was the culprit lesion, so a right guiding catheter was seated to the right coronary ostium. A guidewire was passed to the distal RCA, and 200 *μ*g nitroglycerin was injected into the RCA to evaluate lumen diameter. The lesion was located before the crux, the narrowing proximal to the mid segment of the RCA disappeared, and the chest pain was relieved. The guidewire was removed. The left system was viewed again, and another 200 *μ*g nitroglycerin was injected intracoronarily. A multivessel coronary artery spasm occurred, which was reversed with intracoronary glycerine trinitrate (Figure 3). No percutaneous treatments were done.

## 3. Discussion

Coronary artery spasm is commonly part of the spectrum of atherosclerotic coronary disease [1]. The degree of vasoconstriction during a spasm ranges from clinically undetectable to complete occlusion. Myocardial infarction may ensue in severe or prolonged cases of ischaemia. Multivessel coronary spasm very likely seems to keep a worse prognosis than single vessel spasm [1]. The appearance of coronary vasospastic segments was suitable for stenting. Correct recognition of a spasm is not always easy but percutaneous treatment can be avoided with the proper diagnosis.

Coronary spasm is most often seen clinically in patients about 50 years old and decreases as age advances [2]. Female smokers suffer most frequently from coronary artery spasms [3].

Multiple studies have stated that the lesions at spasm sites have less plaque, no calcification, more diffuse intimal thickening, less lipid and necrotic core, thicker baseline medial width, more prevalent negative remodelling, less thin cap fibrous atheromata, and very small baseline luminal area [4–6]. Meaningful organic coronary artery stenoses are generally low reactive to vasoactive stimuli, presumably because of stiffening of the vessel wall at points of plaque buildup and calcification [7].

A recent study has pointed that the patients with variant angina have additionally not only abnormal reactivity of epicardial coronary arteries but also abnormal coronary microvascular function and peripheral arterial response to vasodilator stimuli [8].

Medical therapy with calcium channel blockers and nitrates is the mainstay of treatment for coronary artery spasm. The use of beta blockers is traditionally avoided given their potential detrimental effect of limiting beta-receptor-mediated dilatation and promoting unopposed alpha adrenergic coronary vasoconstriction [3].

In fact, we did not think of spasm in the patient initially, because of the coronary angiography and FFR reports that documented coronary artery disease and stent implantation in the Cx artery. However, after intracoronary nitrate injection both the right and left coronary system lesions decreased dramatically and dilatation of the coronaries was seen. With a simple intracoronary nitrate injection, unnecessary stent implantation was avoided. Quite possibly, the operators that performed the previous angiographies were not aware of this vasospasm potential in the patient. After angiography, his beta blocker therapy was changed to a calcium channel blocker and nitrate therapy was added.

In patients presenting with ST elevation and no coronary obstruction, there is a high prevalence of myocarditis and thrombophilia, but, in this case, because of coronary artery history and previous hospital examinations there was no reason to think of myocarditis or thrombophilia.

This case report describes an inferior ST elevation on ECG in a patient with existing coronary artery disease. A multivessel coronary artery spasm occurred on coronary angiography during his anginal attack, which emphasises the importance of excluding coronary vasospasm during diagnostic coronary angiography prior to intervention.
